# Duration reproduction with sensory feedback delay: differential involvement of perception and action time

**DOI:** 10.3389/fnint.2012.00095

**Published:** 2012-10-16

**Authors:** Stephanie Ganzenmüller, Zhuanghua Shi, Hermann J. Müller

**Affiliations:** ^1^Department Psychology, General and Experimental PsychologyLMU Munich, Germany; ^2^Graduate School of Systemic NeuroscienceLMU Munich, Germany; ^3^Department of Psychological Sciences, Birkbeck College (University of London)London, UK

**Keywords:** action, audition, time perception, time reproduction, vision

## Abstract

Previous research has shown that voluntary action can attract subsequent, delayed feedback events toward the action, and adaptation to the sensorimotor delay can even reverse motor-sensory temporal order judgments. However, whether and how sensorimotor delay affects duration reproduction is still unclear. To investigate this, we injected an onset- or offset-delay to the sensory feedback signal from a duration reproduction task. We compared duration reproductions within (visual, auditory) modality and across audiovisual modalities with feedback signal onset- and offset-delay manipulations. We found that the reproduced duration was lengthened in both visual and auditory feedback signal onset-delay conditions. The lengthening effect was evident immediately, on the first trial with the onset-delay. However, when the onset of the feedback signal was prior to the action, the lengthening effect was diminished. In contrast, a shortening effect was found with feedback signal offset-delay, though the effect was weaker and manifested only in the auditory offset-delay condition. These findings indicate that participants tend to mix the onset of action and the feedback signal more when the feedback is delayed, and they heavily rely on motor-stop signals for the duration reproduction. Furthermore, auditory duration was overestimated compared to visual duration in crossmodal feedback conditions, and the overestimation of auditory duration (or the underestimation of visual duration) was independent of the delay manipulation.

## Introduction

Accurate timing is essential for our everyday activities, like dancing, playing music, or catching a moving object. In order to accomplish precise timing in a complex environment, our brain has to frequently update its internal representation of multiple sensory inputs. Precisely inferring the timing and duration of events as well as correctly judging temporal order in the sub-second range can be challenging, since neural representations of time may be confounded by noise and delay perturbation in sensory pathways. For example, the neural transmission time can vary across different sensory modalities (King and Palmer, 1985; Regan, 1989), and physical transmission distances (Campbell et al., 1981; Shadmehr et al., 2010), as well as stimulus intensities (Purpura et al., 1990). Continuous changes of the body and the environment provide a further challenge for accurate action timing (Shadmehr et al., 2010). However, in daily life, accurate sensorimotor temporal coordination remains possible, indicating that our brain is able to calibrate and compensate for temporal inconsistencies among different sensory inputs as well as delays in the sensorimotor loop.

Indeed, research has demonstrated that the brain can dynamically realign the perceived timing of multisensory or sensorimotor events. For example, Fujisaki et al. (2004) have shown adaptive changes in synchrony perception between vision and audition: after exposure to a fixed audiovisual asynchrony, the point of subjective simultaneity (PSS, a measure of point in time at which observers perceive maximum simultaneity) of an audiovisual event was shifted toward the previous “lagging” modality. Other work has revealed similar temporal recalibration mechanisms across other modalities (Vroomen et al., 2004; Navarra et al., 2005; Hanson et al., 2008; Harrar and Harris, 2008; Takahashi et al., 2008; Di Luca et al., 2009). Temporal recalibration has also been found between an action and its sensory feedback. The first study that demonstrated compensation for temporal delays in the visuomotor feedback loop confronted participants with a visual-motor lag (delayed visual feedback while controlling the horizontal movement of a small airplane as it moved down the screen through an obstacle field) (Cunningham et al., 2001). Participants' performance improved after some time of practice. Interestingly, when the lag was removed after the adaptation, the adapted behavior persisted and participants, suffering from the adaptation, often made movements too early, leading to more crashes. In another study, Stetson et al. (2006) demonstrated that following brief exposure to delayed visual feedback of a voluntary action the subjective temporal order of a motor-sensory event might even be reversed when the delay was removed. This effect was attributed to dynamical shifts of the appearance of the visual stimulus with respect to the perceived timing of the key press, in order to maintain appropriate causality perception. This proposal goes along with earlier findings that a delayed sensory effect is perceived as having appeared slightly earlier in time if it follows a voluntary action (Eagleman and Holcombe, 2002; Haggard et al., 2002)—a phenomenon referred to as “intentional binding.” Studies have also demonstrated that intentional binding attracts a voluntary action toward its sensory effect, so that the action is perceived as having occurred slightly later in time and the interval between the action and its sensory feedback as shorter than the actual interval (Haggard et al., 2002; Engbert et al., 2007, 2008). Wenke and Haggard (2009) proposed that the shortening effect is driven by a transient slowdown of an internal clock after a voluntary action, and this shortening effect might be reinforced by everyday experience which leads us to assume sensorimotor synchrony between the start of a motor action and its sensory consequence (Heron et al., 2009). However, whether sensorimotor temporal calibration is due to timing changes in the motor system or in the perceptual system is still under debate. Some researchers have suggested that sensorimotor temporal calibration is induced mainly by a temporal shift in the motor system (Sugano et al., 2010), whereas others have attributed sensorimotor temporal calibration to pure perceptual learning (Kennedy et al., 2009).

Alternatively, sensorimotor temporal (re-)calibration has been taken to only reflect modification of predictive feed-forward actions, reducing the errors between the internal prediction and the external feedback (Miall and Jackson, 2006; Shadmehr et al., 2010). Such error correction mechanisms have been used for explaining sensorimotor synchronization, as for instance in the frequently used paradigm of finger tapping to an external pacing source (metronome). When the changes of the pacing source are detectable and regular, participants are able to reduce their sensorimotor asynchronies by predicting upcoming changes. When temporal changes are unpredictable, the time to the next motor response is automatically adjusted in proportion to the asynchrony in the previous sensorimotor event (Repp, 2005).

However, it is important to note that most of the aforementioned studies focused on sensorimotor calibration of a point in time. By contrast, the effects of delayed feedback on the voluntary duration reproduction are as yet little understood. Unlike a point in time, subjective duration can be distorted in many ways, such as by a saccadic eye movement shortly before or after the to-be-estimated event (Morrone et al., 2005), a voluntary action immediately prior to the critical event (Park et al., 2003), the emotional state of the observer (Angrilli et al., 1997; Shi et al., 2012), stimulus properties (such as intensity) (Eagleman, 2008), or pharmacological agents (such as cocaine or methamphetamine) (Meck, 1996) (see review Buhusi and Meck, 2005). Perceived durations in different modalities can also differ. For example, sounds are often perceived as longer than light flashes of the same physical duration (Walker and Scott, 1981; Wearden et al., 1998). Furthermore, there is evidence that the auditory system dominates the visual system, causing the durations of visual stimuli, presented simultaneously with an auditory stimuli, to be perceived as longer than they physically are (Walker and Scott, 1981; van Wassenhove et al., 2008; Burr et al., 2009; Chen and Yeh, 2009; Shi et al., 2010a; Klink et al., 2011). In addition, not only the use of different signal modalities during a timing task, but also the encoding of multiple signal durations, can lead to distortions in temporal memory—an effect recently termed as “memory-mixing” (Gu and Meck, 2011). Such high variability in subjective timing is quite surprising considering how important accurate timing is for our actions.

The purpose of the present study was to investigate how asynchronous-feedback signals would influence motor timing. We adopted an action-based duration reproduction paradigm combined with feedback onset- and, respectively, offset-delay manipulations. That is, participants had to reproduce auditory or visual durations and received (auditory or visual) feedback signals^1^. The feedback could either be synchronized or delayed with participants' button presses (onsets or offsets), and could be delivered in the same or different modality. We specifically asked participants to focus on the reproduction of the standard duration and not pay attention to the feedback. There are two sources of temporal information available for duration reproduction: motor timing (i.e., the duration of the button press) and the feedback timing. If participants only rely on the motor timing for their ongoing reproduction, reproduction errors would be expected to be the same or similar across all trials, no matter whether the feedback is synchronous or delayed. If participants get influenced by the feedback signal during their reproduction, despite the instruction, different reproduction errors for synchronized versus delayed feedback would be predicted. Furthermore, we examined influences of action-effect causal relationship on the duration reproduction, by presenting the feedback signal randomly near the onset or offset of participants' action.

## General methods

### Subjects

Sixty nine naive volunteers (53 females, mean age 27.6) participated in each experiment for payment (Experiments 1–4: 14 participants, Experiment 5: 13 participants). All participants had normal or corrected-to-normal vision; none of them reported any history of somatosensory disorders. They gave written informed consent before the experiments.

### Stimuli and apparatus

All experiments were conducted in a dimly lit cabin (0.21 cd/m^2^). Auditory tones (400 Hz and 600 Hz, 64 dB) and LED lights (84 cd/m^2^ blue and 67 cd/m^2^ red) were presented as stimuli. Stimulus presentation and data acquisition were controlled by a National Instrument PXI system, ensuring highly accurate timing (<1 ms). The experimental programs were developed using MatLab and the Psychophysics Toolbox (Brainard, 1997). The auditory stimuli were delivered to participants via headphones (Pro-luxe XL-300); the LED stimuli (two LEDs, blue and red) were positioned 2 cm apart horizontally. The response button was placed on the table in-between the participant and the LEDs. Reproduction times were measured using the response button, which participants pressed with their right-hand index finger.

### Procedure

**Figure 1:**
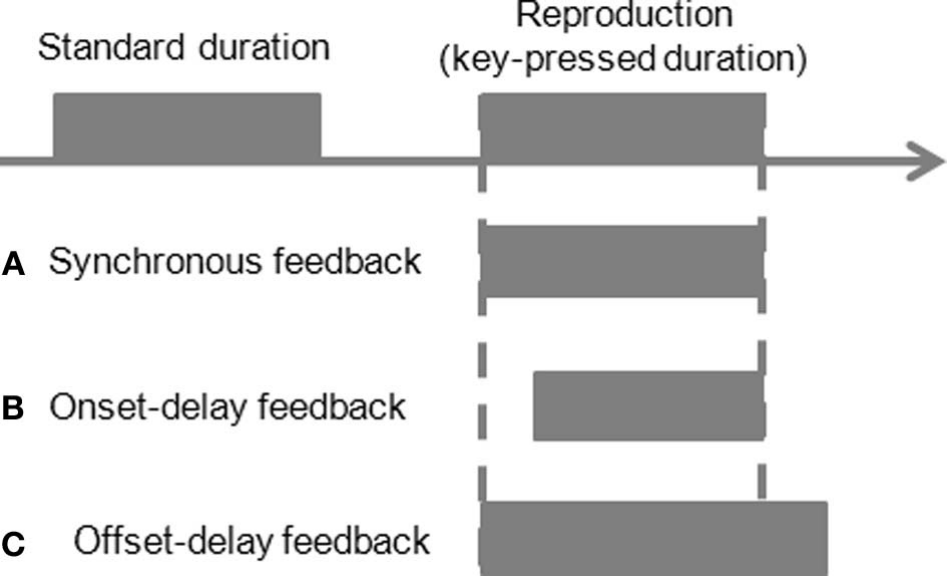
**Schematic illustration of the experimental design.** A standard duration reproduction paradigm with manipulation of feedback delays during reproduction. An auditory or visual stimulus is presented first as a standard duration. Participants reproduce the standard by pressing a button. Another auditory or visual stimulus is fed back to participants based on the action. The feedback signal could be synchronous to the key press (**A** synchronous-feedback condition), or be delayed 200 ms at the onset of the feedback but simultaneously stops at button release (**B** onset-delay feedback condition), or starts synchronously with the button press but stops 200 ms after the button release (**C** offset-delay feedback condition).

We adopted and modified an action-based duration reproduction task with feedback, as introduced by Bueti and Walsh (2010). Each trial started with a standard duration, either 800 or 1200 ms in length, in the form of an auditory tone (Experiments 1 and 4) or an LED light (Experiments 2 and 3). Following the presentation of the standard duration, participants were asked to reproduce the duration as accurately as possible by button press, with reproduction duration demarcated by the onset and offset of the press action. Pressing the button also induced a feedback signal (a tone in Experiments 1 and 3, an LED light in Experiments 2 and 4) whose onset or offset could deviate from the onset or offset of the button press (see Figure 1 and next paragraph). Subjects were told that feedback signal could be either dependent or independent of their button press. They were specifically instructed to reproduce the standard duration as accurately as possible by pressing down the button, regardless of the feedback signals (see the detail instruction in the “Appendix”). To distinguish and counter-balance the standard and feedback stimuli, half of the participants received high tones (or red lights) as standard stimuli and low tones (or blue lights) as the ± feedback stimuli, and vice versa for the other half.

For the first four experiments, there were three different temporal manipulations of feedback signals: synchronous-feedback, onset-delay feedback, and offset-delay feedback. In the synchronous-feedback condition, the onset and offset of the feedback occurred synchronously with the onset of the button press and the release of the button. In the onset-delay condition, the onset of the feedback signal was delayed by 200 ms following the onset of the button press, while feedback offset occurred synchronously with the release of the button. In the offset-delay condition, the feedback signal started synchronously with the button press, but the feedback offset occurred only 200 ms after the release of the button. These three conditions were varied block-wise, with 10 trials per block. Both the onset- and offset-delay blocks were preceded and followed by a synchronous-feedback block. The order of the onset- and offset-delay blocks was randomized.

In Experiment five, we used the same block-design as in previous experiments, but randomized the onset and offset of the feedback signal relative to the button press. To do this, for each synchronous-feedback block we measured the mean reproduction durations for 800 and 1200 ms, and the mean response onset asynchrony. During the onset-manipulation blocks, the feedback signal started independently of the button press, with random jittering ±200, ±100, or 0 ms around the mean response onset asynchrony measured in the preceding synchronous block. The feedback signal stopped when the button was released. During the offset-manipulation blocks, the feedback signal started synchronously with the button press, but stopped automatically with a duration randomly jittering ±200, ±100, or 0 ms around the mean reproduction duration (either 800 or 1200 ms corresponding to the duration in the current trial) measured in the preceding synchronous block. The random jittering was used in order to ensure that participants would not be able to predict the onset or offset of the manipulated feedback signal, thus we could obtain about half of all trials with feedback prior to participants' actions. We further increased the number of the trials to 20 for the onset- and offset-manipulation blocks to ensure enough trials with the feedback before participants' action. The task instruction was kept the same as during the previous four experiments.

Note that the standard and feedback stimuli were kept within the same modality in Experiments 1, 2, and 5, but presented in separate modalities in Experiments 3 and 4 (see Table 1).

**Table 1:** **Modalities of the standard and feedback stimuli**.

**Experiment**	**Standard**	**Feedback**
1	Auditory	Auditory
2	Visual	Visual
3	Visual	Auditory
4	Auditory	Visual
5	Auditory	Auditory

In the first four experiments, there were 10 repetitions for the onset- and offset-delay blocks and 20 repetitions for the synchronous-feedback signal blocks. Participants took a short break after every eight blocks. In Experiment 5, there were eight repetitions for the onset- and offset-manipulation blocks (each consisting of 20 trials) and 16 repetitions for the synchronous-feedback signal blocks (each consisting of 10 trials). Here, participants took a short break after four blocks (= 60 trials). In addition, there were two practice blocks with the synchronous-feedback signal condition run prior to the formal experiment.

### Data analysis

Mean measures and standard deviations of time reproduction have been shown to vary linearly with standard durations, so that after normalization the same form of distribution of relative time and constant timing sensitivity can be found (Gibbon et al., 1984). In line with this, reproduction errors (i.e., the difference between the reproduced duration and the standard duration) in the present study exhibited differences between the two standard durations (800 and 1200 ms), that is, the amount of over-/underestimation (in ms) is proportional to the respective standard duration. To take this into account, we calculated reproduction errors and then normalized them by the corresponding physical duration. Normalized reproduction errors of zero indicate perfect reproduction, positive values an overestimation, and negative values an underestimation of the standard duration. In order to examine dynamic influences of the onset- and offset-delay manipulation, we selected four trials from the synchronous block prior to and the synchronous block after the delay manipulation. The first four trials served as baseline and the last four trials for analyzing after-effects of the delay manipulation. Henceforth, we refer to the former four synchronous-feedback trials as baseline phase, the latter four synchronous-feedback trials as post phase, and the 10 trials from the (intervening) delay block as delay phase. We omitted the middle two trials in the synchronous-feedback block to separate the post and baseline phases. Repeated-measures analyses of variance (ANOVAs) of the normalized reproduction errors in the three different phases (baseline phase, delay phase, and post phase) were run separately for the onset- and offset-delay conditions. Bonferroni-corrected *t*-tests for multiple comparisons were carried out for a-posteriori comparisons to assess differences in reproduction errors.

For Experiment 5, we focused on analyzing linear correlations between the onset- and offset-manipulations and normalized reproduction errors. Thus, linear regression and correlation analyses were applied. We realigned the onsets of the feedback relative to the onsets of the actual response, and compared the differential influences between the feedback before and after participants' action. For the offset-manipulation condition, we used an alternative approach: we calculated the offset jitters relative to the standard durations and analyzed the general relationship between the offset jitters and the reproduction errors. We did not align the offsets relative to the responses, since the mean feedback duration was close to the mean reproduction time, which would inevitably lead to pseudo negative correlation between the relative offset and the reproduced duration. Such correlation could not reflect the influence of the offset-manipulation. In both cases, we normalized feedback jitters with their correspondent standard durations, such that the feedback jitter has the same unit as the normalized reproduction error.

## Results

### General reproduction results

**Figure 2:**
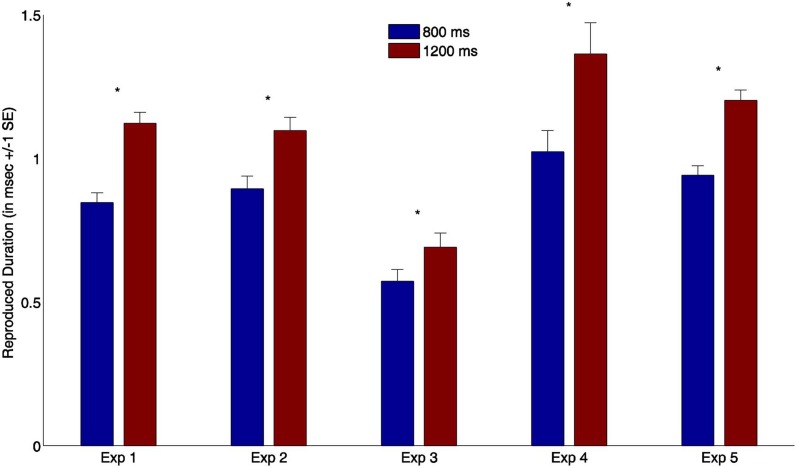
**Mean reproduction times (and associated standard errors) from all synchronous trials for all five experiments.** Blue bars depict produced durations after short standard stimuli (800 ms); red bars indicate reproduction times after long standard stimuli (1200 ms) (^*^ indicates *p* < 0.01).

We analyzed reproduction times for the synchronous-feedback condition for all five experiments, comparing reproduction performance after the short (800 ms) and long (1200 ms) standards. Reproduced durations in milliseconds are presented in Figure 2. We found a significant difference between the reproduced times of the short and long standard stimuli (all *p* < 0.01) across all five experiments, suggesting participants were actually able to perform the task.

### Effects of unimodal feedback onset- and offset-manipulation on the duration reproduction

**Figure 3:**
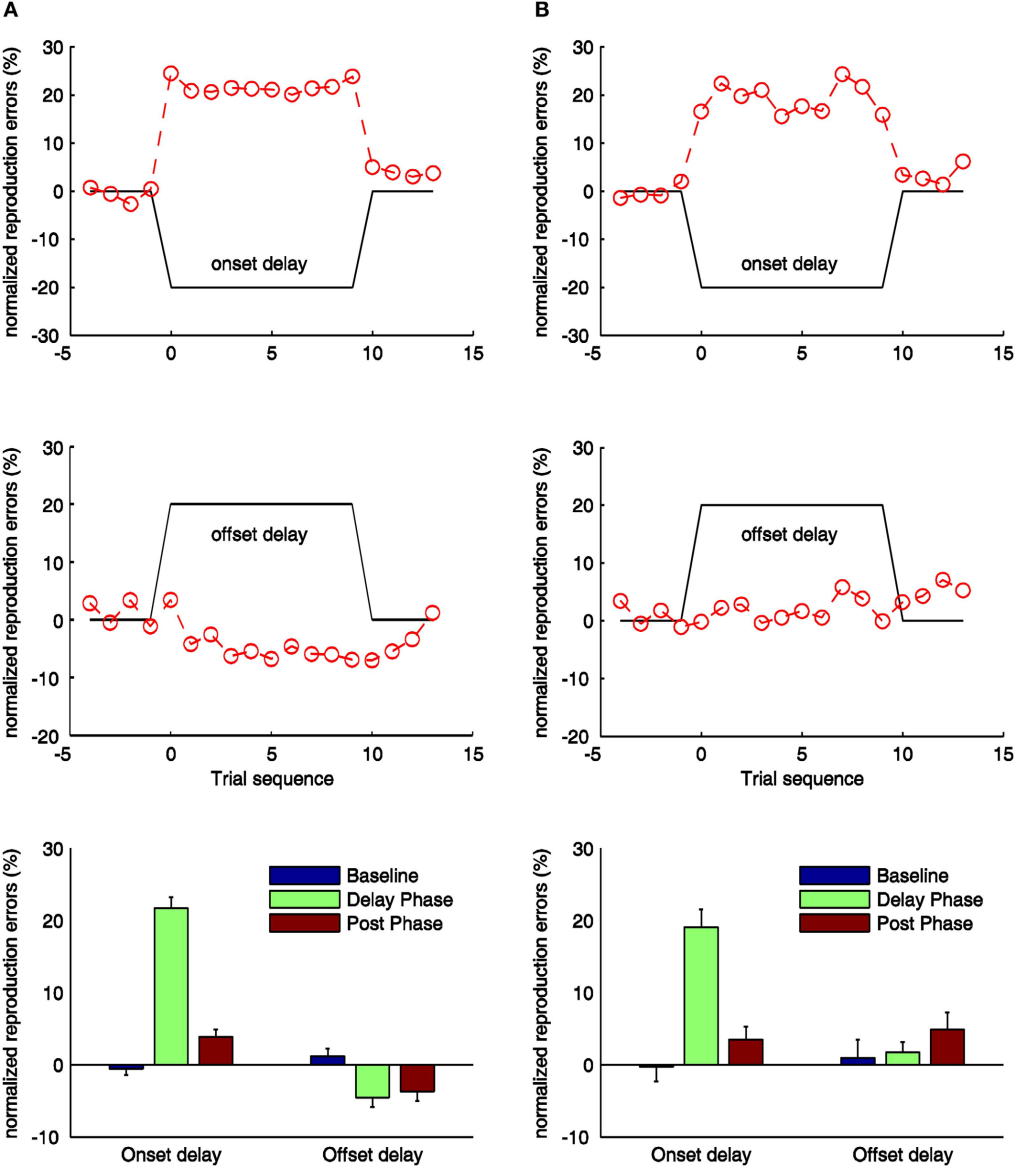
**Normalized reproduction errors [(subjective duration—physical duration)/physical duration] for the onset- and offset-delay condition of Experiment 1 (A) and Experiment 2 (B).** In the upper and middle panels trial-wise dynamic changes of normalized reproduction are shown. Four trials from the synchronous block before the delay manipulation (baseline phase), delay block (delay phase), and four trials after the delay manipulation (post phase) are displayed. The black lines indicate the physical delay. The red dashed curves and circles depict mean normalized reproduction errors as a function of trial sequence and the onset-delay (up-panel) or offset-delay (middle panel). In the low-panels mean normalized reproduction errors (and associated standard errors) are plotted against baseline, delay and post phase for the onset- and offset-delay conditions.

Normalized reproduction errors, and associated standard errors, for the first four experiments and all conditions are presented in Table 2. Figure 3 shows the normalized reproduction errors for the onset- and offset-delay manipulation for the unimodal auditory and visual feedback.

**Table 2:** **Normalized reproduction errors (± standard errors) in percentage by onset- and offset-delay manipulation and different phases in Experiments 1–4**.

	**Onset-delay manipulation**			**Offset-delay manipulation**		
	**Baseline phase**	**Delay phase**	**Post phase**	**Baseline phase**	**Delay phase**	**Post phase**
Experiment 1	−0.55 ± 2.5	21.73 ± 2.0	3.89 ± 2.8	1.18 ± 2.9	−4.57 ± 1.8	−3.69 ± 2.9
Experiment 2	−0.28 ± 4.4	19.09 ± 2.7	3.51 ± 4.4	0.95 ± 4.3	−1.72 ± 2.8	4.91 ± 4.2
Experiment 3	−33.88 ± 3.1	−12.16 ± 2.2	−31.48 ± 3.4	−33.06 ± 3.4	−37.93 ± 2.1	−38.19 ± 3.6
Experiment 4	21.01 ± 4.6	37.21 ± 3.3	24.39 ± 5.3	22.55 ± 4.9	23.35 ± 3.4	25.47 ± 5.6

In the onset-delay conditions (Figure 3, up-panels), normalized reproduction errors were significantly influenced by the delay manipulation, [*F*_(2, 26)_ = 246.78; *p* < 0.01], and [*F*_(2, 26)_ = 43.30, *p* < 0.01] for the auditory and visual conditions respectively. The overestimation during the onset-delay phase for both auditory and visual conditions proved to be significantly larger compared to the baseline (*p* < 0.01) and the post phase (*p* < 0.01) (Figure 3, low-panels). Normalized reproduction errors in the post phase (overestimation) were raised reliably relative to the baseline (*p* < 0.01) for the auditory condition, but not for the visual condition (*p* = 0.16). Interestingly, the overestimation on the onset-delay phase was 21% for the auditory and 19% for the visual, which are statistically not different from the onset-delay manipulation (all *p* > 0.1). Furthermore, the overestimation started with the first trial of the delay manipulation (condition) and stopped as soon as the delay was removed (Figure 3, up-panels). Paired *t*-tests showed no significant difference in the overestimation between the first versus the remaining trials in both delay and post phase, (all *p* > 0.1).

In contrast to the onset-delay manipulation (which made participants overestimate the standard durations), the offset-delay manipulation (Figure 3, mid-panels) showed different patterns for the auditory and visual conditions. In the auditory condition (Figure 3, middle left panel), the offset-delay led participants to significantly underestimate the standard durations during the offset-delay phase, [*F*_(2, 26)_ = 13.73; *p* < 0.01]. This effect derived mainly from a significantly negative increase in normalized reproduction errors during the delay phase versus the baseline (*p* < 0.01). Normalized errors were also negatively increased in the post phase compared to the baseline (*p* < 0.01). However, there was no reliable difference between the delay and post phases (*p* = 0.99). Paired *t*-tests showed that the underestimation started only from the second trial with delay manipulation, as there was no effect in the first trial of the delay phase (significant difference between the first and the remaining trials, [*t*_(13)_ = 9.30, *p* < 0.01]). Also, underestimation only stopped on the second trial of the post phase, with reproduction errors on the first trial still differing significantly from the errors on the other trials, [*t*_(13)_ = −5.26, *p* < 0.01]. In contrast to the auditory condition, manipulation of the visual offset-delay feedback had no significant influence on normalized reproduction, [*F*_(2, 26)_ = 1.60, *p* = 0.22] (baseline vs. delay: *p* = 1.00; delay vs. post phase: *p* = 0.36; baseline vs. post phase: *p* = 0.45).

## Effects of crossmodal feedback onset- and offset-manipulation on duration reproduction

**Figure 4:**
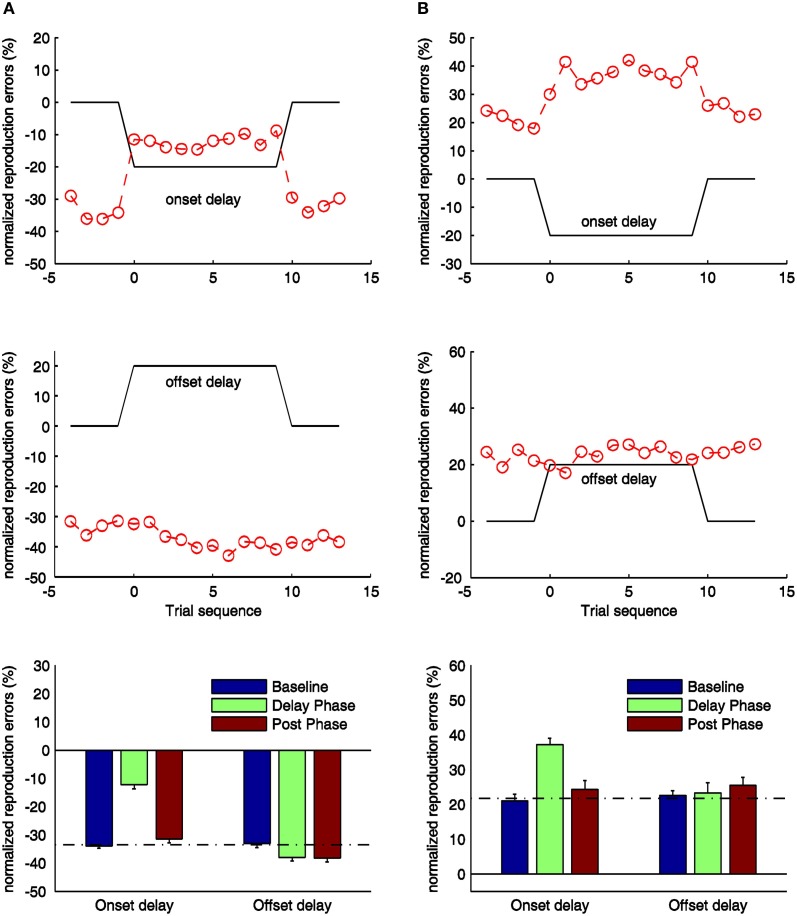
**Normalized reproduction errors for the onset- and offset-delay condition of Experiment 3 (A) and Experiment 4 (B).** In the upper and middle panels trial-wise dynamic changes of normalized reproduction are shown. Four trials from the synchronous block before the delay manipulation (baseline phase), delay block (delay phase), and four trials after the delay manipulation (post phase) are displayed. The black lines indicate the physical delay. The red dashed curves and circles depict mean normalized reproduction errors as a function of trial sequence and the onset-delay (up-panel) or offset-delay (middle panel). In the low-panels mean normalized reproduction errors (and associated standard errors) are plotted against baseline, delay, and post phase for the onset- and offset-delay conditions. The dashed line indicates the mean normalized reproduction error in the baseline condition.

Overall, there was strong underestimation of the visual standard with synchronous auditory feedback signal (hereafter we refer to as the visual-auditory experiment), and strong overestimation of the auditory standard with visual feedback signal (hereafter the auditory-visual experiment), all *p* < 0.01. Trial-wise normalized reproduction errors for the onset- and offset-delay manipulations are depicted in Figure 4.

For the onset-delay conditions (Figure 4, up-panels), the normalized reproduction errors were significantly modulated by onset-delays for the visual-auditory experiment, *F*_(2, 26)_ = 185.41, *p* < 0.01, and the auditory-visual experiment, *F*_(2, 26)_ = 39.06, *p* < 0.01. The underestimation (in the visual-auditory experiment, Figure 4A) and the overestimation (in the auditory-visual experiment, Figure 4B) in the onset-delay phase, were significantly different from the correspondent baseline and the post phase (all *p* < 0.01), while there were no differences between the baseline and post phase (all *p* > 0.1). Interestingly, the reproduced duration during the onset-delay phase compared to the baseline was increased 21% for the visual-auditory experiment and 16% for the auditory-visual experiment. Both are comparable to the overestimation observed in Experiment 1 and 2 (21 and 19% respectively). Further pair-wise sequential-trial analysis showed that the manipulation effect of the onset-delay in the visual-auditory experiment started on the first trial of delay manipulation (*p* = 0.78) and stopped as soon as the delay was removed (*p* = 0.28). However, in the auditory-visual experiment, participants needed one trial to adjust their behavior to the onset-delay, as evidenced by significantly different normalized reproduction errors in the first trial compared to the remaining trials of the delay phase, *t*_(13)_ = −2.57, *p* < 0.05. However, the effect ceased as soon as the delay was removed (*p* = 0.59).

For the visual-auditory experiment, a general, significant underestimation was also found in the offset-delay condition, *F*_(2, 26)_ = 8.15, *p* < 0.01 (Figure 4A, mid-panel). Relative to the baseline, the normalized reproduction error (underestimation) was negatively increased in the offset-delay phase (*p* < 0.05) and in the post phase (*p* < 0.05); there was no difference between the latter two phases (*p* = 1.00). The increased underestimation due to the offset-delay manipulation is again comparable to the results of Experiment 1. Sequential-trial analysis revealed both the first and the second trial to differ significantly from the remaining trials in the delay phase [first: *t*_(13)_ = 2.58, *p* < 0.05; second: *t*_(13)_ = 5.03, *p* < 0.01]. In the post phase, normalized reproduction errors did not change over trials (*p* > 0.1). Trial-wise comparisons of delay- and post-phase reproduction errors yielded no significant differences (all *p* > 0.1). Thus, participants either needed more than four trials to readjust their reproduction performance to the synchronous-feedback, or normalized reproduction errors were too variable within trials. However, for the auditory-visual experiment, the offset-delay manipulation did not influence the reproduction performance, *F*_(2, 26)_ = 0.95, *p* = 0.40. None of the phases differed from any other (all *p* > 0.1). This result is similar to that obtained in Experiment 2.

### Effects of random onset- and offset-manipulation on the duration reproduction

**Figure 5:**
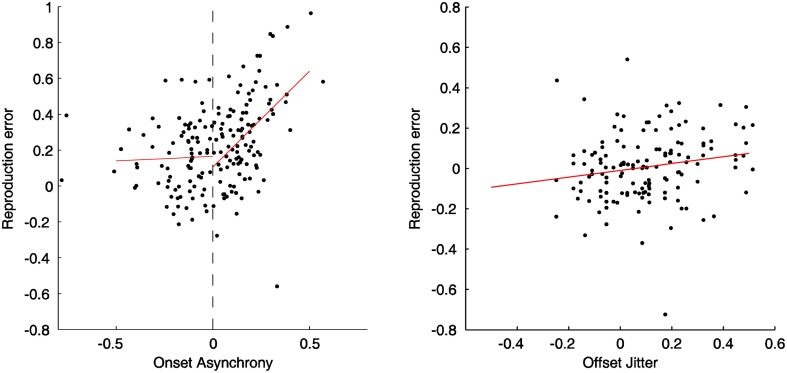
**Normalized reproduction errors and linear regression lines (red) for the onset (left side) and offset (right side) manipulation conditions from a typical dataset.** In the onset-manipulation condition, the fitted slope for the feedback signal started before the action onset (0.06) is not significant different from zero, while the slope for the delayed feedback (1.07) is significant higher than zero. In the offset-manipulation condition the slope is 0.30, significantly higher than zero.

Figure 5 illustrates relationships between the reproduction error and the relative feedback onset (left panel) and offset (right panel) for a typical participant. For the onset-manipulation condition, there was a significant correlation between positive feedback delays and reproduction errors (correlation coefficient: 0.41, linear slope: 0.89, all *p* < 0.05). The steep slope indicates an about 89% compensation for the delayed onset in the duration reproduction, which was similar to the finding in Experiment 1. However, such correlation was broken down when the feedback was presented before participants' actions. There was no correlation [mean: 0.1, *t*_(12)_ = 0.81, *p* = 0.43] for those “preceded” feedback trials, and the mean slope (0.17) did not significantly differ from zero, *t*_(12)_ = 0.90, *p* = 0.39. For the offset-manipulation condition, the correlation between reproduction errors and random offsets was mildly related, mean correlation coefficient 0.31, *t*_(12)_ = 6.53, *p* < 0.05. The mean slope (0.3) was significant higher than zero, *t*_(12)_ = 8.31, *p* < 0.05, though it was significantly lower than the mean slope of the “delayed” onset condition, *t*_(12)_ = 3.83, *p* < 0.05. The mild offset modulation confirmed the findings in Experiments 1 and 3.

## Discussion

The results of the present study illustrate how the onset- and offset-manipulation of the feedback signal influences the duration reproduction. In all experiments, we found an increase in duration reproduction for conditions with positive onset-delay feedback manipulation. The lengthening of the reproduced duration could almost compensate the onset-delay (about 90% for the auditory feedback and 75–90% for the visual feedback). The subjective lengthening started immediately with the first trial (or second in Experiment 4), and ended with the last trial of the delay phase. Despite our explicit instruction for reproducing the standard duration regardless the feedback signal, the reproduced duration was still heavily influenced by the onset of the delayed feedback. However, such influence was broken down when the feedback signal was presented before participant's button press.

The results suggest that the action-effect causal relationship may play a critical role in the duration reproduction. Through prior experience, we have learnt that the effect of an action is not always immediate (Pesavento and Schlag, 2006). For example, the response of a tap on the computer keyboard becomes visible as a letter on the screen only after a delay of some 20–50 ms, and the response of a remote control might even be slower (Rank et al., 2010; Shi et al., 2010a,b; Sugano et al., 2010). The action-effect causal relationship may lead to bind and recalibrate motor-sensory timing (Cunningham et al., 2001; Stetson et al., 2006), to attract a voluntary action toward its sensory effect (Haggard et al., 2002; Engbert et al., 2007, 2008), and to shift attention toward to the sensory feedback (Buehner and Humphreys, 2009). Such causal binding may well relate to the memory-mixing model (Gu and Meck, 2011). Due to limited capacity of working memory and the cause-effect relationship, motor timing, and *caused*-feedback timing may share the same representation, which pulls both onsets closer. Other studies have also shown similar binding and regression effects in the reproduction task (Teghtsoonian and Teghtsoonian, 1978; Lejeune and Wearden, 2009; Jazayeri and Shadlen, 2010). For example, participants are able to use temporal context (such as mean duration) to reduce variability of their performance by sacrificing accuracy during a reproduction task (Lejeune and Wearden, 2009; Jazayeri and Shadlen, 2010). However, when the causal relationship is violated (i.e., the feedback was prior to the action in Experiment 5), linkage between two events—the action and sensory feedback—becomes weak, which leads to less memory interference between the two representations. The causal binding and memory-mixing could also explain the quick adjustment to the onset-delay, since the binding and immediate adjustment of the reproduction can take place in the same trial.

In contrast to the effects of introducing feedback onset-delays, offset-delay manipulation appears to modulate duration reproduction in a modality-dependent manner, though with comparatively small effects. Duration reproduction for the auditory offset-feedback delay (Experiments 1, 3, and 5) was shortened by only some 25–30% of the delay manipulation, while there was no shortening effect for the visual offset-delay manipulation. The latter was probably due to sluggish visuomotor timing (Jäncke et al., 2000; Repp, 2005). With the auditory offset-delay manipulation, the shortening effect became manifested not on the first trial with a delay, but only on the second or third trial. Similarly, the shortening effect diminished more gradually after the removal of the delay (after one trial in Experiment 1 and probably more than four trials in Experiment 3). This dynamic adaptation is comparable to previously observed adaptive changes in synchrony perception (Fujisaki et al., 2004; Vroomen et al., 2004). Also, the amount of adaptation (25% of the auditory offset-delay manipulation) resembles previously reported shifts in PSEs for point-in-time calibration [e.g., 10% for multisensory adaptation (Fujisaki et al., 2004; Di Luca et al., 2009), and 29% for sensorimotor adaptation (Sugano et al., 2010)]. The partial compensation has been attributed to the fact that the brain takes into account a long history of “veridical” sensory inputs throughout lifetime, as compared to only a short adaptation phase during typical psychophysical experiments (Fujisaki et al., 2004). Similar in our study, the asynchrony between the end of an action and the end of the *auditory* feedback may be used as an error signal (Shadmehr et al., 2010) for sensorimotor adaptation to partially adjust future actions. As suggested by the memory-mixing account (Gu and Meck, 2011), participants may use the representation of previous experienced offset-delay for predicting a potential delay on a given offset-manipulation trial.

Mild partial compensation also suggests that participants trust their own stop signal more than the delayed offset signal. This may relate to the switch of the internal clock model (Gibbon, 1977; Gibbon et al., 1984), which consists of a pacemaker emitting pulses at a certain rate and a mode switch that can open and close to permit an accumulator to collect emitted pulses. When the switch closes, the number of pulses in the accumulator is compared against a reference time from memory. Larger amounts of accumulated pulses mean longer estimated durations. Recent striatal beat-frequency (SFB) model provides a neurobiological plausible model of interval timing and switch (Matell and Meck, 2004), which suggests timing is based on the coincidental activation of medium spiny neurons in the basal ganglia by cortical neural oscillators. At trial onset the synchronization of cortical oscillators is triggered by the dopaminergic burst, and at expected offset a burst is reflected on cortico-striatal transmission (see review Buhusi and Meck, 2005). It has been shown that neurons in the motor cortex increase their synchrony when animals are trained to expect an action (Riehle et al., 1997). The synchronization triggered by the expected stop-action might be considered as the more reliable switch-off signal than the offset of the external sensory feedback, leading to the offset-delay interval being largely neglected and to less memory-mixing than during the onset condition. This could also explain the findings in Experiment 5, where the feedback offset was random and unreliable.

In Experiments 3 and 4, in which the standard duration and the feedback signal were presented in different modalities, we observed a strong distortion of perceived durations: visual standard durations were strongly underestimated by presentation of auditory feedback signals during the reproduction, and this finding was mirrored by a strong overestimation of auditory standard durations when the feedback signal was a visual stimulus. The over- and underestimations across the audiovisual modalities are analog to previous findings. For example, Wearden et al. (1998) have provided evidence that the auditory pacemaker ticks faster than the visual pacemaker, as a result of which auditory durations are perceived as longer than physically equivalent visual durations. However, it remains an open question whether the observed audiovisual effects are mainly caused by the crossmodal memory-mixing. Nevertheless, recall that the overestimation (underestimation) was additive to the effects of delay manipulation, which suggests that the crossmodal standard-feedback signals comparison (i.e., presenting a standard stimulus in one modality and providing a feedback signal stimulus in another modality) is operating mainly on the perceptual level, relatively independent of sensorimotor adjustments.

## Conclusion

In summary, the present study investigated the effects of feedback signal delay manipulation on active duration reproduction. When the onset of sensory feedback signals was delayed, reproduced durations lengthened immediately to compensate for the feedback signal delays in large proportion. The feedback before action onset was neglected. However, when the offset of sensory feedback signals was delayed, reproduced durations only shortened by about 25–30% of the delay with auditory feedback signals, while there was no compensation for visual feedback signals. These results suggest that active duration reproduction is heavily mixed with the delayed feedback onset and mildly influenced by the feedback offset. The results can be explained with causal binding and the memory-mixing accounts. Moreover, the observed under- and overestimation due to crossmodal manipulation of the standard and feedback signal stimuli is additive to the sensorimotor delay adaptation.

### Conflict of interest statement

The authors declare that the research was conducted in the absence of any commercial or financial relationships that could be construed as a potential conflict of interest.

## Footnotes

^1^In this study we refer to the second stimulus—that is presented during the reproduction—as a “feedback signal” to highlight the causal relationship between the action and sensory effect. The terms “feedback signal” and “feedback” are used interchangeably in the text.

## Appendix

### Instruction

In this experiment your task is to reproduce the duration of a tone by pressing a button. For each trial, you will first hear a tone for a certain duration. Please try to memorize the temporal information as accurately as possible! As soon as the tone stops, you are asked to press the button in front of you for as long as you heard the tone before. It is important for the experiment that you reproduce the duration of the first tone as accurately as possible! While you press the button, another tone will be presented. This tone could be either dependent or independent of your button press. Therefore, please try to reproduce the duration of the first tone, regardless of the second tone! There will be a practice block in the beginning for familiarization with the task. After the practice block the actual experiment will be started automatically. There will be 10 blocks for the whole experiment, which lasts about 45 min.
